# Physician Consultations According to Different BMI Levels of the Greek General Population

**DOI:** 10.3390/ijerph8114300

**Published:** 2011-11-14

**Authors:** Evelina Pappa, Nick Kontodimopoulos, Angelos A. Papadopoulos, Yannis Tountas, Dimitris Niakas

**Affiliations:** 1 Hellenic Open University, Faculty of Social Sciences, Riga Fereou 169 & Tsamadou, Patras 26222, Greece; E-Mails: nkontodi@otenet.gr (N.K.); docpapado@yahoo.gr (A.A.P.); niakas@eap.gr (D.N.); 2 “ATTIKON” University Hospital, Rimini 1, Athens 124 62, Greece; 3 Center for Health Services Research, Department of Hygiene and Epidemiology, Medical School, Athens University, Alexandroupoleos 25, Athens 115 27, Greece; E-Mail: chsr@med.uoa.gr

**Keywords:** obesity, overweight, physician consultations, health needs, Greece

## Abstract

Obesity constitutes a global epidemic which is rapidly becoming a major public health problem in many parts of the world, threatening peoples’ health and quality of life. The aim of our study was to estimate the prevalence and impact of overweight and obesity on physician consultations and frequency of use and furthermore, to investigate whether physician consultations in each of the groups defined by BMI level correspond to the need for care implied by health risk level, using logistic regression models. The survey was carried out in Greece in 2006 and involved complete data from 645 individuals consulted by physicians. Overweight and obese users constituted 41.7% and 19% of the sample respectively. The findings showed firstly that the odds of obese individuals visiting a physician (OR 2.15) or making more than three visits (OR 2.12) was doubled compared to the odds of individuals with normal weight. Secondly, we conclude that physician consultations in overweight and obese subgroups as well as the frequency of visits were predicted by factors such as co-morbidities, low HRQL, low educational level which are associated directly or indirectly with obesity, and thus with a greater health need, assuming vertical equity in the utilization of such services.

## 1. Introduction

According to the WHO [1] obesity constitutes a global epidemic, which is rapidly becoming a major public health problem in many parts of the world, threatening peoples’ health and quality of life. Both overweight and obesity are associated with increased mortality and especially with increased morbidity and disability and require long-term management with emphasis on prevention. The prevalence of overweight and obesity is increasing worldwide, resulting in an escalation of health care use and further economic burden.

In Greece, the variation of the prevalence of overweight and obesity by gender is evident. According to a recent study [2] with a national representative sample the prevalence of overweight and obese were 41% and 26% in men and 20% and 18% in women, whereas in another study [3] were 53% and 20% in men and 31% and 15% in women. Unfortunately, the scarcity of epidemiological data precludes us from observing longitudinal prevalence. However, excess body weight is reaching epidemic proportions in Greece and obesity rates are among the highest in Western countries [2].

Excess body weight increases the risk for many disorders and a large body of studies has shown that many co-morbid conditions, including diabetes mellitus, cardiovascular disease, stroke, hypertension, and certain cancers have been associated with obesity [4–9]. Furthermore, obesity and overweight were associated with decreased health-related quality of life (HRQL) [10–12]. On the other hand, the relationship between socio-economic status and excess body weight has been widely investigated, and has been generally shown to be inversely related to obesity, with evident gender differences [10,13–16].

The association between BMI and health care utilization is another issue of great importance. In health service utilization studies the impact of factors such as health need indicated by health status, demographic and socioeconomic characteristics is well-documented describing the degree of equity in the use of primary health care services. Hence, it is equally important to investigate the relationship between obesity/overweight and health service utilization, where potential confounding variables (demographic, socioeconomic, co-morbidities, *etc*.) can be controlled for. To date, we are unaware of any study in Greece having addressed the impact of overweight and obesity on health care use. However, in international bibliography the impact of both obesity and overweight on primary health service utilization has been studied intensively [17–22] in respect to frequency of use and health care costs and results have demonstrated that excess body weight is associated with increased use of health services and expands the cost of health care. Excess body weight with its accompanying disorders places individuals in different health risk groups, implying that it is important to investigate the association between BMI and primary setting services such as physician consultations.

Our objective in this study was threefold. Firstly, to record utilization in the primary sector and especially of physician consultations by BMI class during one year period. Secondly, to quantify the impact of obesity and overweight on physician consultations compared to normal weight. Thirdly to investigate if, controlling for potential confounding variables such as socio-demographic characteristics and health need factors, results in physician consultations corresponds to the need for care defined by obesity health risk levels. The rest of the paper is organized as follow: we define the study design, then we describe the estimation methods, further the results are presented and finally we conclude with some discussion.

## 2. Methods

### 2.1. Study Design

The cross-sectional study took place in September 2006 and involved a sample of adults (>18 years old) residing in urban (>2000 inhabitants) and rural (<2000 inhabitants) areas of the country and in each of the 13 geographical regions. According to the latest Population Census (2001), the survey population consisted of approximately 8,880,924 individuals. Non-fluent Greek speakers, institutionalized subjects and those incapable of reasoning and decision-making on their own were excluded. Participants were grouped, in proportion to the Greek population, by socio-demographic characteristics, according to a three-staged sampling methodology. In the first stage, a random sample of building blocks was selected in proportion to size. In the second stage, households were randomly selected by systematic sampling. In the third stage, an eligible participant was selected by simple random sampling in each household. In total 1005 willing subjects, out of 1388 initially approached (response rate 72.4%), were interviewed by trained interviewers. The present study involves a subsample of 645 individuals having consulted physicians and for whom complete data were available.

### 2.2. Measures

Participants were asked to report their height and weight. BMI is the predominant index for identifying obesity and classifies individuals into three weight classes: (i) 18.5–24.9 kg/m^2^ (normal), (ii) 25–29.9 kg/m^2^ (overweight) and iii) BMI ≥30 kg/m^2^ (obese) [1]. The dependent variables were defined firstly as those who had at least one physician contact during a one year period according to BMI and secondly by frequency of contacts as 1–2 visits *vs* 3 or more. Furthermore, socio-demographic, health needs and health risk factors relating to lifestyle were used as possible explanatory variables. Demographics included gender, age and marital status. Education, according to three levels, (primary, secondary and university) and employment (employer, employee, retired, other) were used as socioeconomic status indicators Participants also reported their place of residence (urban/rural).

Health needs were proxied by self-assessed health, hospital admissions and co-morbidity. Self-assessed health was measured by the Greek version of SF-12 which has been validated with a national representative general population sample in a previous study [23], and was used to measure physical and mental health of the respondents on a scale of 0–100, with higher scores reflecting better perceived physical and mental health. Co-morbidity was based on the presence and the number of chronic diseases. Respondents were asked to report whether they suffered from at least one of the following thirteen chronic conditions: diabetes mellitus type I & II, hypertension, dyslipideamia, heart failure, coronary ischemic disease, irritable bowel syndrome, chronic bronchitis, asthma, osteoarthritis, Alzheimer’s, depression and anxiety disorders. Participants were also asked to report any hospital admissions during the previous twelve months. Any use (admission) of hospital services was contrasted to no use (no admissions). Finally, an individual’s harmful lifestyle was reflected by smoking and alcohol consumption. Participants were classified as non *vs* daily/occasionally smokers. Alcohol consumption was determined by the portions (e.g., glasses of wine) of alcoholic beverages consumed typically in a week, with respondents classified as consuming up to seven glasses of wine per week *vs* more.

### 2.3. Statistical Analysis

Chi-square test was used to assess the prevalence of physician consultations and frequency of visits according to different levels of BMI. Additionally, we used odds ratio (OR) as a measure of the effect of obesity on physician consultations. Logistic regression analyses were applied to identify the parameters that determine the two decision processes theoretically involved in health care utilization, *i.e.*, the decision to seek care (initiated by patient/user) and the frequency of visits. Multinomial logistic regression models, using forward stepwise selection, were conducted to determine whether physician consultations in each BMI group were predicted by health need, as implied by the health risk level of the BMI group each user belong to. A binary logistic regression model using forward Wald selection was further applied and we used the exponentiation of the B coefficient Exp(b) in order to estimate the adjusted odds ratio (OR), with 95% confidence intervals, of the frequency of visits according to levels of obesity (overweight and obese), controlling for socio-demographic and health need factors. Results were considered statistically significant when *p* < 0.05 and all analyses were performed using SPSS v17.0.

## 3. Results

Complete data was available for 645 individuals conducting physical consultations. BMI scores ranged from 17.58 to 46.87 kg/m^2^, the mean and median BMI were 26.4 and 26.1 kg/m^2^ respectively (data not shown for parsimony), implying that the study sample was, on average, overweight. Socio-demographic characteristics and health variables according to BMI level are presented in Table 1. Two hundred sixty nine (41.7%) respondents were classified as overweight and approximately nineteen percent (*n* = 122) of the individuals were obese (15.7% men and 21.4% women). It is worth mentioning that men were more prone to overweight whereas women were to obesity. Normal weight decreased with age, whereas overweight and obesity increased. Married individuals and low educational level (primary) were more probable to overweight and obesity. As it was expected, obese individuals suffered more from chronic diseases than the rest of the groups. On the other hand, obesity or overweight did not appear to be related to hospitalization or polypharmacy (four or more drugs) or even more so with unhealthy habits such as smoking or high alcohol consumption.

Physician consultations and their frequencies are presented in Table 2, and it is evident that both are positively associated with BMI level. The proportion of obese individuals initiating a physician consultation is very high (79.7%), with three or more visits having been reported by 64.8% of the obese users (*p* < 0.05).

With respect to the effect of excess body weight on primary care use, we estimated the odds ratio (OR) for physician consultations and frequency of visits (Table 2) for overweight and obese compared to normal weight which was the control group. The results showed that the odds of overweight subjects to consult a physician was 11.8% (OR: 1.18) the odds of the normal weight subjects, whereas the odds of obese subjects to consult a physician was more than two times (OR: 2.15) the odds of the control group. A similar pattern was observed for frequency of visits. The odds of overweight subjects to make three or more visits was 12.3% (OR: 1.23) the odds of the normal weight subjects whereas the odds of obese subjects to make three or more visits was more than two times (OR: 2.12) the odds of normal weight subjects.

### 3.1. Use of Physician Consultations

Table 3 displays the adjusted odds ratio of physician consultations among overweight or obese *vs* normal weight users. For the overweight users subgroup, men were more likely to be overweight (OR: 3.0, CI: 1.96–4.56). Being in a marriage increased the likelihood of being overweight (OR: 3.2, CI: 1.90–5.29) by approximately three times. Primary educational level (OR: 3.7, CI: 1.97–6.81) or the existence of chronic diseases (OR: 2.2, CI: 1.34–3.54) increased the probability of being an overweight user.

The odds of being an obese user of physician services were for married individuals two and a half times the odds of singles (OR:2.5, CI: 1.25–4.99), respectively. Individuals with primary education (OR: 3.4, CI: 1.51–7.13) or individuals reporting at least one chronic disease (OR: 2.4, CI: 1.32–4.36) were more likely to be obese users compared to individuals with university education or those reporting no chronic diseases respectively. On the other hand better physical health (OR: 0.97, CI: 0.94–0.99) decreased the odds of consulting a physician by 3%.

### 3.2. Frequency of Consultations

In investigating the association between body weight and number of consultations (Table 4), the results of the unadjusted logistic regression model have shown that obese users were more than twice as likely to have made three or more visits to a physician (OR: 2.2, CI: 1.42–3.46), whereas the effect of overweight on the number of visits was not statistically significant (*p* > 0.05). In the adjusted logistic regression model, and after controlling for other socio-demographic and health need factors, the odds of having three or more consultations with a physician were predicted only by health needs factors, and the effect of BMI became statistically insignificant. More specifically, users that were hospitalized within the last year (OR: 1.92, CI: 1.06–3.49) or reporting having at least one chronic disease (OR: 1.8, CI: 1.13–2.80) had a higher likelihood of making three or more visits, whereas having better physical and mental health (OR_PCS-12_: 0.95, OR_MCS-12_: 0.97) decreased the probability of making three or more visits.

## 4. Discussion

### 4.1. Main Findings and Comparisons with Other Studies

Our study had three objectives. First, we wanted to record the prevalence of physician consultations, according to BMI categories. The results have shown that physician consultations as well as their frequency are high within each BMI category and increased with obesity. Two reasons might explain our findings. First, the fact that the rate of physician consultations in the total sample was high (64.2%), a finding which has been also reported in other studies [18]. Secondly, the high consultation rate of the obese population, in relation to the fact that the majority of obese users (67%) have made three or more visits during a one year period imply that the obese population are heavy users of primary care and impose an additional burden on the health care sector in terms of impaired health. Indeed, previous studies have shown that obesity is associated with two or more co-morbidities [24], increased risk of disability [25] and decreased HRQL [10,12] while in terms of more physician visits implying greater health care costs [26].

In an attempt to quantify the impact of overweight and obesity on physician consultations, which was the second objective, odds ratio estimates have shown a moderate impact of overweight on both physician consultations (11.8%) and frequency of visits, *i.e.*, three or more visits (12.3%) despite the statistical insignificance. As for obesity, the results have shown a significant impact, with the odds of consulting a physician and of making three or more visits were two times higher the odds of normal weight users. The increased burden on health care use by obese subjects is interpreted by many confounding factors that are associated with obesity and furthermore with poor health conditions. The existence of co-morbidities is a significant factor which can lead to further morbidity and mortality [27,28]. Age is another risk factor of developing obesity and at the same time is associated with many medical conditions. Obesity is also associated with disadvantaged socioeconomic status *i.e.*, low educational level, as our results have shown and other studies have confirmed [10,15], which is in term related to lower health knowledge [29].

Our third objective confirmed the fact that physician consultations correspond to the need for care, as defined by the risk group of the individuals. Overweight and obese were more likely to consult a physician than those with normal weight. Overweight users were more likely to be men, married, having primary education and suffering from chronic diseases, whereas obese users were more likely to be married, having primary education, suffering from chronic diseases, and having impaired physical health. Multinomial regression analysis -controlled for age- has shown that the primary cause of increased use of physician contacts by overweight and obese was poor health status due to the presence of co-morbidities, poor HRQL (specifically physical health) and low educational level which has been extensively-documented to be associated with poor health status [30,31].

Greater health need and therefore poorer health status as implied by the three confounding factors is associated with greater use, assuming the existence of vertical equity in the relationship between BMI and primary health care use. A particularity that could explain both the high rate of physician consultations and the existence of vertical equity is the structure of the Greek public primary health care sector, in which access is free through insurance funds and the NHS. Another significant confounding factor was marital status. As previous studies have shown, marital status influences the likelihood of developing overweight and obesity [32], or changes in social roles such as marriage influence physical characteristics such as body weight [33].

Concerning frequency of visits, we noted that in an unadjusted model obesity was associated with three or more visits, but in an adjusted model where other confounding variables were included, no association between BMI and frequency of visits was observed. Our results were not in accordance with previous study [34] which had shown that obesity was associated with the mean number of primary care visits, diagnostic services and clinic charges. Our results suggest that the association between BMI and frequency of use is more a function of the relationship between BMI and impaired health status as determined by co-morbidities, hospitalization and HRQL.

A point that requires consideration is the inclusion or not of subjects with obesity-related co-morbidities such as diabetes 2, hypertension, *etc*. in the analysis (whether obesity generates higher health care use) as this might lead to biased outcomes. Previous studies have chosen two different approaches. The first approach [35] proposed the exclusion of subjects with certain co-morbidities which can lead to an over-adjustment of the statistical control for such diseases because they were thought as intermediates along the causal pathway between increased BMI and increased health care use [36]. According to the second approach [19] obesity-related co-morbidities were included and SF-36 physical health was used as a surrogate for co-morbid conditions. In our study we used both approaches and the results remained the same, suggesting that obesity-related co-morbidities did not bias the results, and that the second approach presented by Bertakis [19] is more appropriate. Of course further research is needed.

### 4.2. Limitations

A limitation of our study is the fact that the weight and height data are self-reported. It is known that self-reported weight and height tend to be underestimated leading in an underestimation of the “real” BMI (objectively measured) [37] and the prevalence of overweight and obese, further resulting in an underestimation of the OR and the association between BMI and health service use. However, the majority of studies use BMI as an obesity measure which is recommended by WHO.

## 5. Conclusions

The results of our study provide important information regarding the impact of increased physician consultations and the factors that determine the use according to different levels of BMI. We conclude that the major cause of increasing health care use of overweight and obese individuals is health need. The existence of co-morbidities, impaired physical and mental health and low educational level were the direct and indirect factors that structure the greater health need of overweight and obese users. From a policy perspective, the additional burden that overweight and obese impose on the health care system, concurrently increases the workload of physicians, as the majority of the Greek population visit primary care physicians, who have an important role in tackling obesity effectively. The strength of influence of individuals’ characteristics on physician consultations, distinguished by obesity, and the increased burden on the health care system reinforces the need for prevention strategies against factors that contribute to the development of obesity.

## Figures and Tables

**Table 1:** Socio-demographic distribution of the sample according to BMI categories.

	Normal (*n* = 254, 39.4%)		Overweight (*n* = 269, 41.7%)		Obese (*n* = 122, 18.9%)	
	
	*n*	%	*n*	%	*n*	%
**Gender**						

Men	94	33.6	142	50.7	44	15.7
Women	160	43.8	127	34.8	78	21.4

**Age**						

18–24	52	73.2	14	19.7	5	7.0
25–34	60	56.1	26	33.6	11	10.3
35–44	42	40.4	48	46.2	14	13.5
45–54	40	41.2	32	33.0	25	25.8
55–64	21	21.4	47	48.0	30	30.6
65+	39	23.2	92	54.8	37	22.0

**Marital status**						

Single	89	63.6	37	26.4	14	10.0
Married	165	32.7	232	45.9	108	21.4

**Education**						

Primary	47	21.6	113	51.8	58	26.6
Secondary	136	45.2	117	38.9	48	15.9
University	69	55.6	39	31.5	16	12.9

**Occupation**						

Employers	30	32.6	44	47.8	8	19.6
Employees	89	47.8	79	42.5	18	9.7
Retired	46	25.8	90	50.6	42	23.6
Other	89	47.3	55	29.3	44	23.4

**Residence**						

Urban	204	42.3	193	70.7	85	72.0
Rural	50	30.7	76	46.6	37	22.7

**Chronic Diseases**						

Yes	58	23.3	122	49.0	69	27.7
No	165	51.6	115	35.9	40	12.5

**Hospitalization**						

Yes	28	30.1	45	48.4	20	21.5
No	224	41.1	219	40.2	102	18.7

**Medication use**						

0	154	51.9	106	35.7	37	12.5
1–3	89	34.0	119	45.4	54	20.6
4+	11	13.1	42	50.0	31	36.9

**Smoking**						

Yes	115	44.1	110	42.1	36	13.8
No	139	32.6	159	41.4	86	22.4

**Alcohol**						

Up to 7/daily	215	39.4	221	40.6	36	20.0
8+	39	39.0	48	48.0	13	13.0

*n* = sample size.

**Table 2:** Use, frequency of use and odds ratio (OR) estimates according to BMI.

	Normal		Overweight		Obese	
	
	*n*	%	*n*	%	*n*	%
**Physician consultation**^a^						

Yes	245	63.8	269	68.3	122	79.7
No	139	36.2	125	31.7	31	20.3
sig.	*p* < 0.05					
**OR**			1.18		2.15	
sig			*p* > 0.05		*p* < 0.001	

**Frequency of visits**						

1–2	137	53.9	131	48.7	43	13.8
3+	117	46.1	138	51.3	79	64.8
sig.	*p* < 0.05					
**OR**			1.23		2.12	
sig.			*p* > 0.05		*p* < 0.001	

a Physician consultation referring to the initial sample of 1005 individuals according to BMI, categorizing those having or not physician contact, in order to indicate the prevalence of use in each BMI category. sig = statistical significant at the level of *p* < 0.05.

**Table 3:** Factors predicting physician consultations according to multinomial logistic regression analysis.

Variables	Overweight (*n* = 269)			Obese (*n* = 122)		

	OR	95% CI	*p*-value	OR	95% CI	*p*-value
Gender (men)	3.0	1.96–4.56	<0.0001	1.4	0.84–2.40	NS
Marital status (married)	3.2	1.90–5.29	<0.0001	2.5	1.25–4.99	0.010
Education (university)						
Primary	3.7	1.97–6.81	<0.0001	3.4	1.51–7.13	0.003
Secondary	1.5	0.92–2.64	NS	1.6	0.77–3.18	NS
Chronic disease (no)	2.2	1.34–3.54	0.002	2.4	1.32–4.36	0.004
PCS-12	1.01	0.98–1.03	NS	0.97	0.94–0.99	0.023
Log likelihood			1.196E3			
Chi-square			134.285 (*p*-value < 0.0001)			
Pseudo *R*^2^ (Nagelkerke)			0.239			

PCS-12 = Physical Component Score. NS = non significant (*p* > 0.05). For categorical explanatory variables, the reference group for the calculation of the odds ratio (OR) is indicated in the parenthesis. CI = Confidence Intervals.

**Table 4:** Frequency of use (1–2 visits *vs.* 3 or more visits) according to logistic regression models.

Variables	Stage 1 (unadjusted model) a			Stage 2 (adjusted model) a		

	OR	95% CI	*p*-value	OR	95%	CI *p*-value
BMI (normal)						

Overweight	1.2	0.88–1.75	NS	0.8	0.52–1.23	NS
Obese	2.2	1.42–3.46	<0.001	0.9	0.55–1.64	NS
Age				1.0	0.98–1.02	NS
Hospitalization (no)				1.9	1.06–3.49	0.032
Chronic disease (no)				1.8	1.13–2.80	0.012
PCS-12				0.95	0.93–0.97	<0.001
MCS-12				0.97	0.95–0.99	0.024
Log likelihood		667.519				
Chi-square		110.887 (*p*-value <0.001)				
Pseudo *R*^2^ (Nagelkerke)		0.236				
Observation		569				

a Stage 1 = unadjusted model and stage 2 = adjusted for other confounding socio-demographic and health need variables. PCS-12 = Physical Component Score, MCS-12 = Mental Component Score. NS = non significant (*p* > 0.05). For categorical explanatory variables, the reference group for the calculation of the odds ratio (OR) is indicated in the parenthesis. CI = Confidence Intervals.
